# Supplementary material for: DNA double-strand break repair pathway regulates PD-L1 expression in cancer cells
Source: Nat Commun. 2017 Nov 24;8:1751. doi: 10.1038/s41467-017-01883-9 (PMC5701012; doi:10.1038/s41467-017-01883-9)
Supplement: Supplementary file 1 — Supplementary Information [file 41467_2017_1883_MOESM1_ESM.pdf]

Supplementary Figure 1

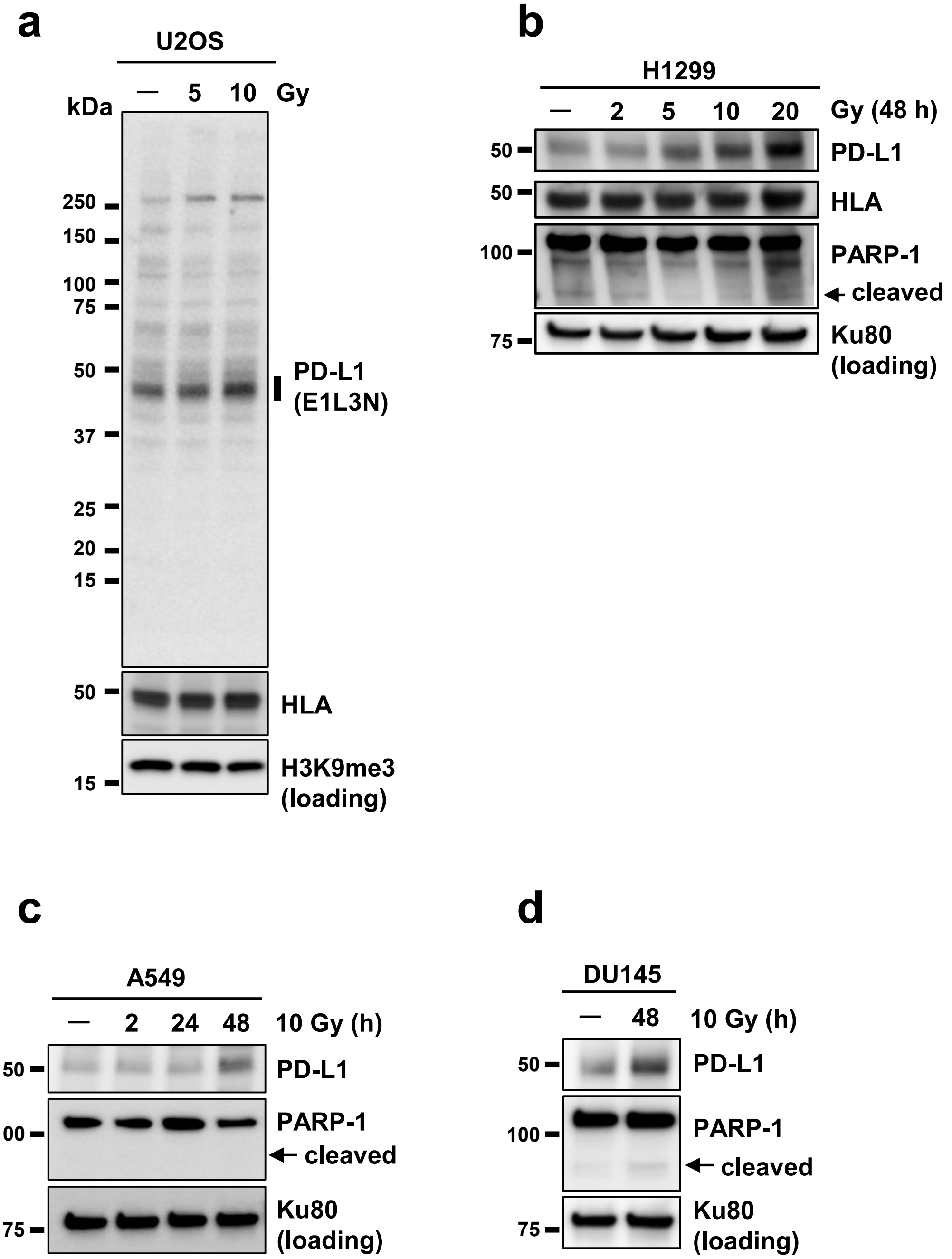

Supplementary Figure 1. PD-L1 expression is induced in response to DNA damage

a) Full-membrane blotting of PD-L1 is shown. U2OS cells were harvested 48 h after IR. HLA level was not significantly altered at 48 h after IR.

b-d) Upregulation of PD-L1 was similarly observed in the H1299 (b), A549 (c), and DU145 (d) cells after IR.

Supplementary Figure 2

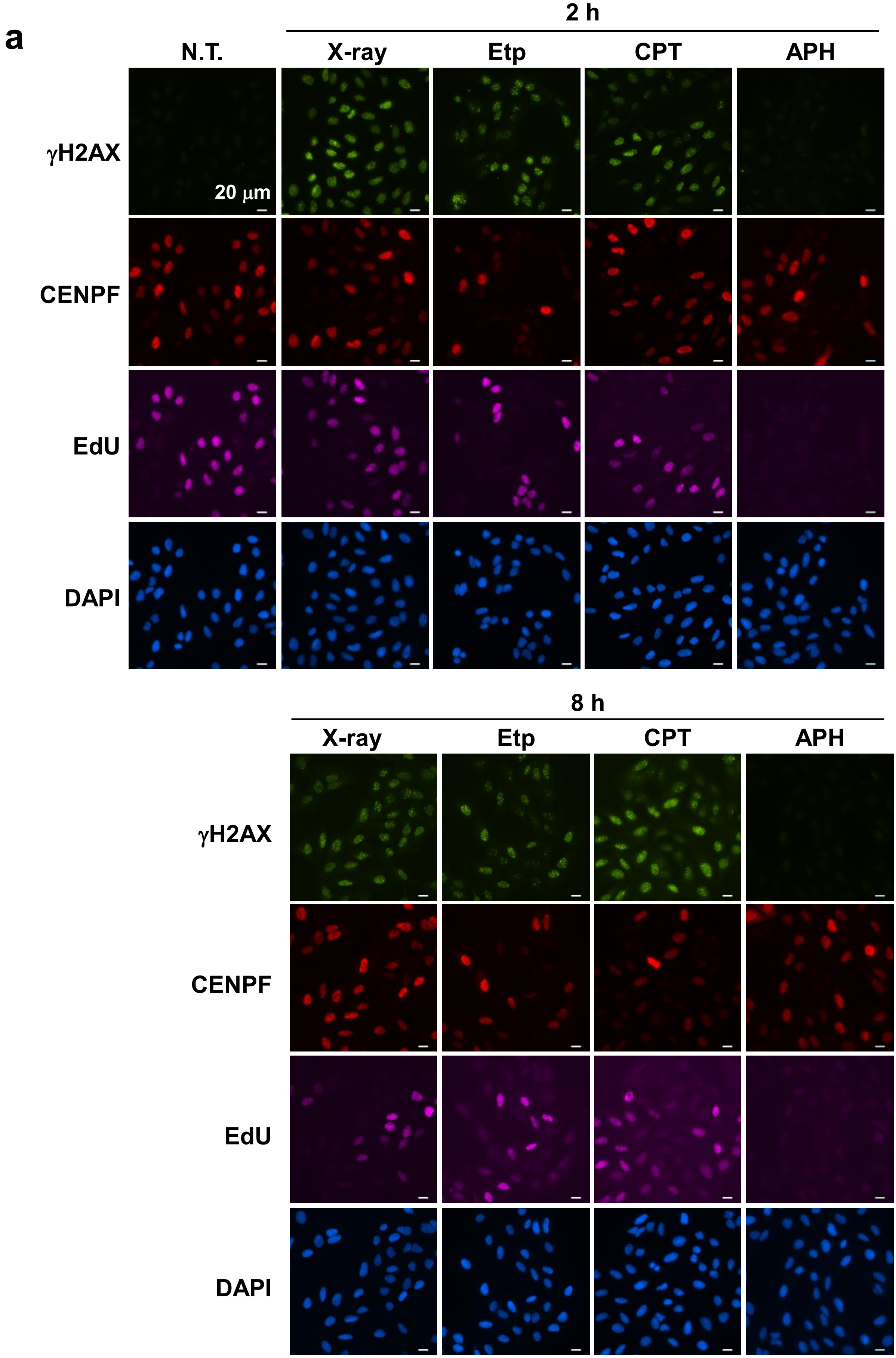

Supplementary Figure 2

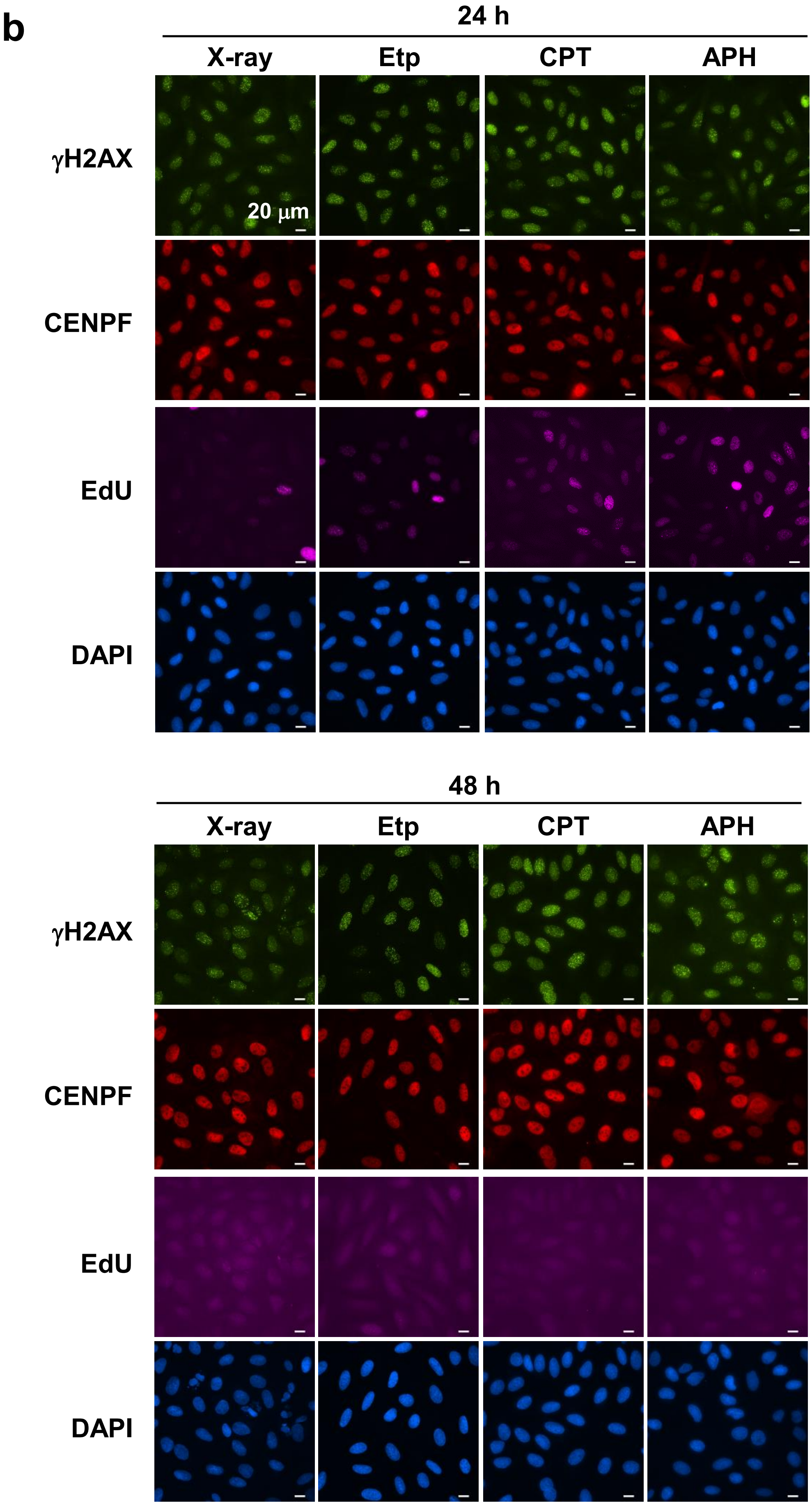

Supplementary Figure 2

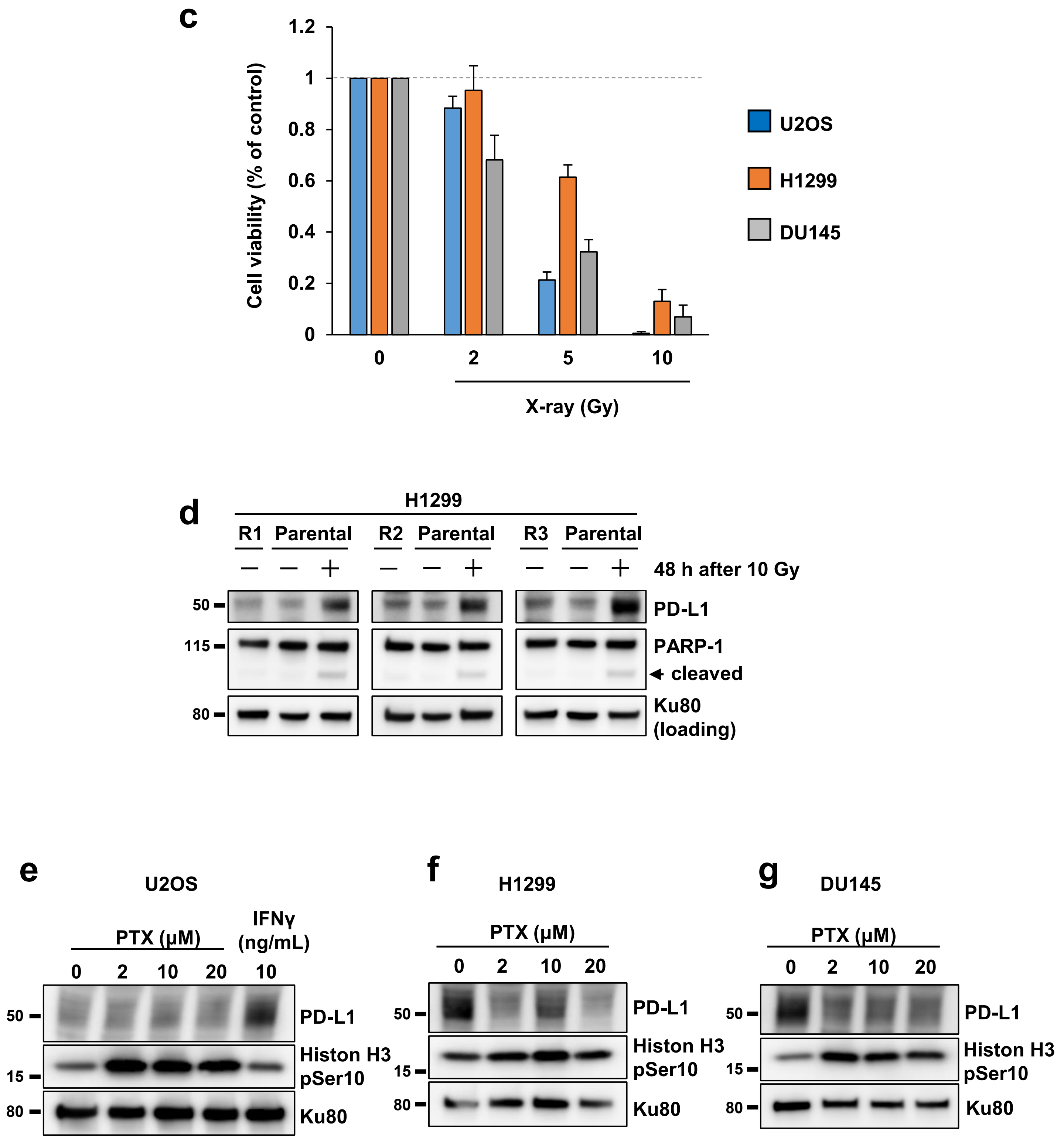

Supplementary Figure 2. Analysis of DNA damage and cell viability after DNA damage

a, b) U2OS cells were irradiated at 10 Gy X-ray or were treated with 500 nM Etp, 50 nM CPT and 500 nM APH. Cells were fixed at indicated time point and stained with γH2AX, CENPF, EdU and DAPI. To identify S phase cells, EdU was added prior to DNA damage. In addition, to identify G2 cells, cells were stained with CENPF. EdU+/CENPF+ cells are in the S phase, and EdU-/CENPF+ cells are in the G2 phase. The scale bar represents 20 μm.

c) Colony formation assay was performed in U2OS, H1299 and DU145 cells after IR. Cells were fixed at 14 days after IR.

d) The IR-induced PD-L1 upregulation were not maintained in surviving cells. H1299 cells were irradiated with 10 Gy. Following the incubation for >14 days after IR, an individual colony (R1, R2 and R3) was picked up. R1–R3 cells were harvested and PD-L1 expression levels were examined. Un-irradiated H1299 cells and 10 Gy-irradiated H1299 cells (48 h) were used as a control. Hence, as shown by the colony assay, the majority of 10 Gy irradiated cells eventually die or stop growing within ~14 days. However notably, the PD-L1 expression levels return back to normal in surviving cells.

e-g) PD-L1 expression was examined in U2OS (e), H1299 (f) or DU145 (g) cells at 48 h after paclitaxel (PTX) treatment. The accumulation of mitotic cells by PTX was confirmed by histone H3 pSer10, a marker of mitosis.

Error bars represent the s.d. of three independent experiments (c).

Supplementary Figure 3

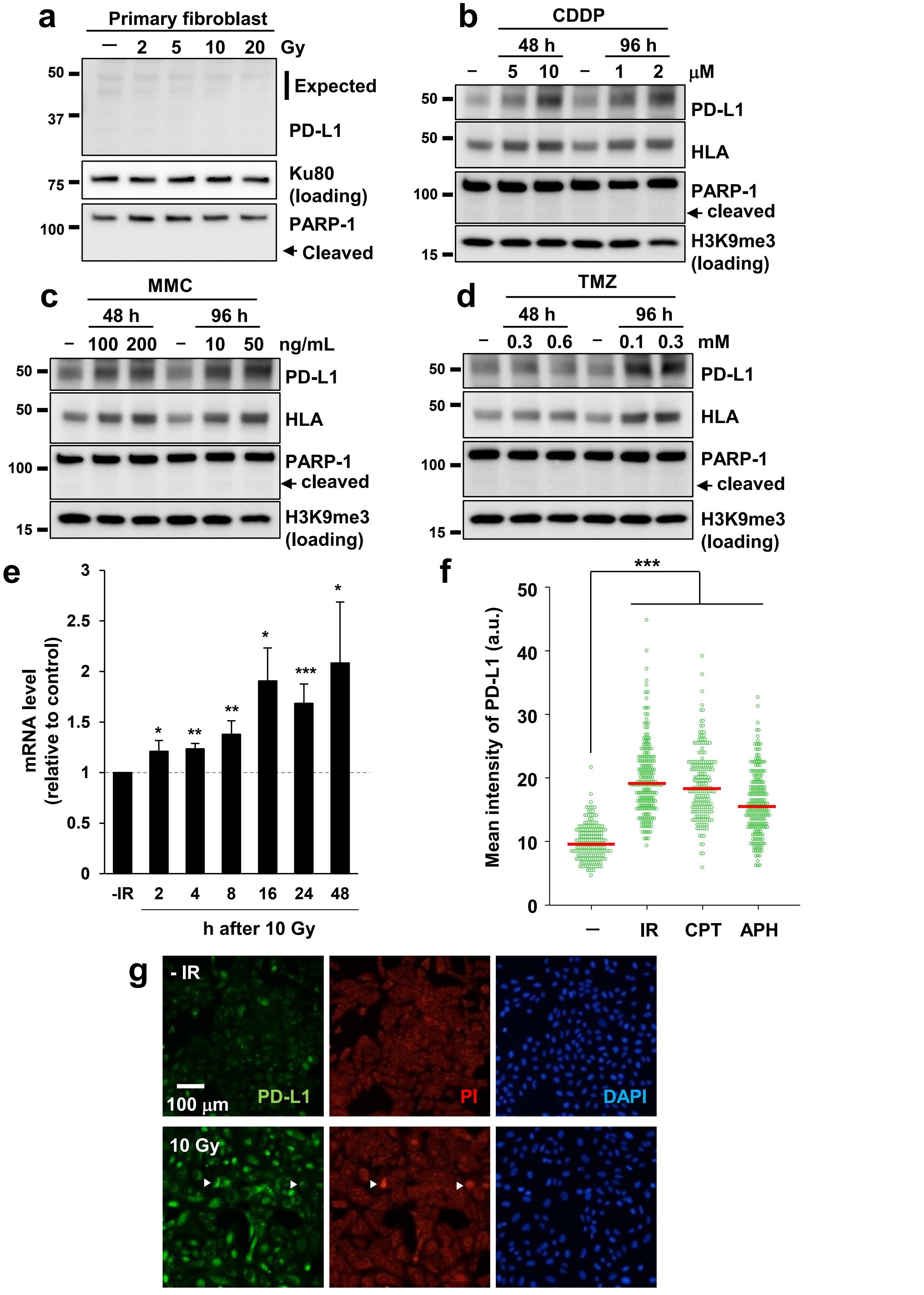

Supplementary Figure 3. Examination of PD-L1 expression after DNA damage

a) IR does not effectively upregulate PD-L1 in human primary fibroblast cells. 48BR (WT) primary fibroblast cells were harvested 48 h after IR. The expression might be distinctively regulated between cell lines. Thus, PD-L1 expression after DNA damage in other normal cell line or organs will be investigated in the future.

b-d) Treatment with chemotherapeutic drugs, which induce DNA damage, upregulates PD-L1. U2OS cells were treated with cisplatin (CDDP: DNA cross-linking agent), mitomycin C (MMC: DNA cross-linking agent) or temozolomide (TMZ: alkylating agent). Cells were harvested at the indicated time points.

e) PD-L1 mRNA levels at 2–48 h after 10 Gy were measured by qPCR. Statistical significance was examined compared with non-treated cells.

f, g) Cell-surface PD-L1 in irradiated U2OS cells was examined by immunofluorescence without permeabilisation. Mean intensity of PD-L1 per cell, except for the DAPI-positive area, was measured by ImageJ 1.48v. Dead cells identified by PI (white arrowheads) were excluded from the analysis. A representative image is shown in panel g. Similar results were obtained in more than two independent experiments.

Error bars represent the s.d. of three independent experiments (e).

Supplementary Figure 4

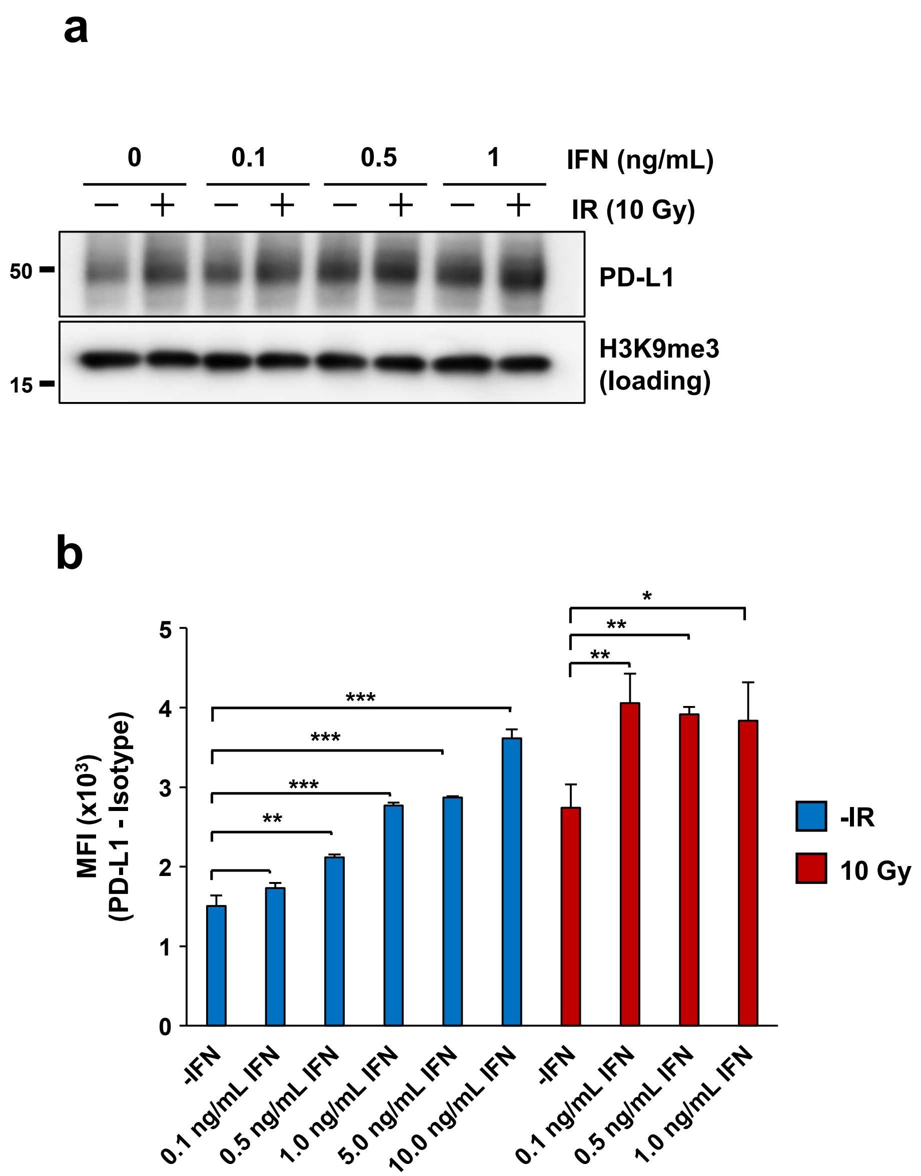

Supplementary Figure 4. Analysis of IR-induced PD-L1 upregulation in the presence of IFNγ
a) IR induces an additive PD-L1 upregulation in the presence of IFNγ. An indicated amount of IFNγ was added 30 min prior to IR. U2OS cells were harvested 48 h after irradiation at 10 Gy.
b) IR induced PD-L1 expression +/- IFNγ treatment; it was examined by FACS. U2OS cells were harvested 48 h after 10 Gy +/- IFNγ. U2OS cells treated with IFNγ alone were harvested 24 h after the treatment. In this experiment, we did not observe a synergistic PD-L1 upregulation by IR in the presence of IFNγ. However, it might be caused in other cell lines or *in vivo*.
Error bars represent the s.d. of three independent experiments (b).

Supplementary Figure 5

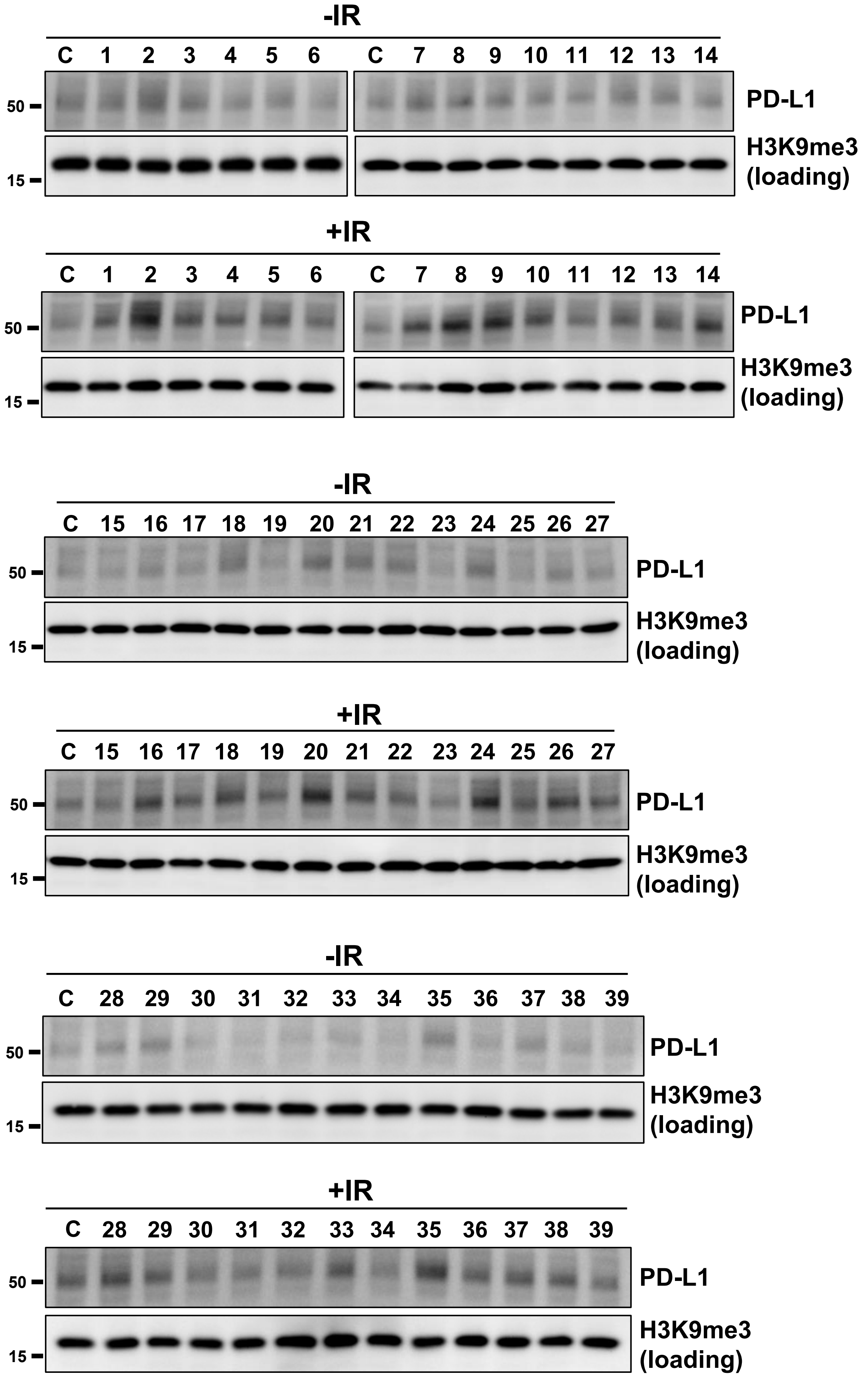

Supplementary Figure 5

NHEJ

1. DNA-PK
2. Ku80
3. XLF
4. LIG4
5. PAXX
6. XRCC4

HR

7. BRCA1
8. BRCA2
9. PALB2
10. BRCC3
11. RAP80
12. RBBP8
13. BARD1
14. ABRA1
15. RAD1
16. RAD10
17. RAD18
18. RAD21
19. RAD23A
20. RAD23B
21. RAD51
22. RAD51C
23. RAD51L1
24. RAD51L3
25. RAD52
26. RAD54B
27. RAD54L
28. XRCC2
29. XRCC3
30. SMC1A
31. SMC3
32. SMC5
33. SMC6
34. Artemis
35. DNA2
36. EXO1
37. EXD2
38. MUS81
39. GEN1

Supplementary Figure 5. Analysis of PD-L1 expression with or without IR following siRNA library of DSB repair genes

U2OS cells exposed to siRNA (ON-TARGETplus siRNA) were harvested 48 h after irradiation at 10 Gy. PD-L1 expression was examined by immunoblotting. The signal intensity of PD-L1 and H3K9me3 was quantified by ImageJ 1.48v. After normalisation of the PD-L1 signal using the loading control H3K9me3, the relative PD-L1 signal for each siRNA compared with that of siControl was determined, as shown in Fig. 3a, b.

Supplementary Figure 6

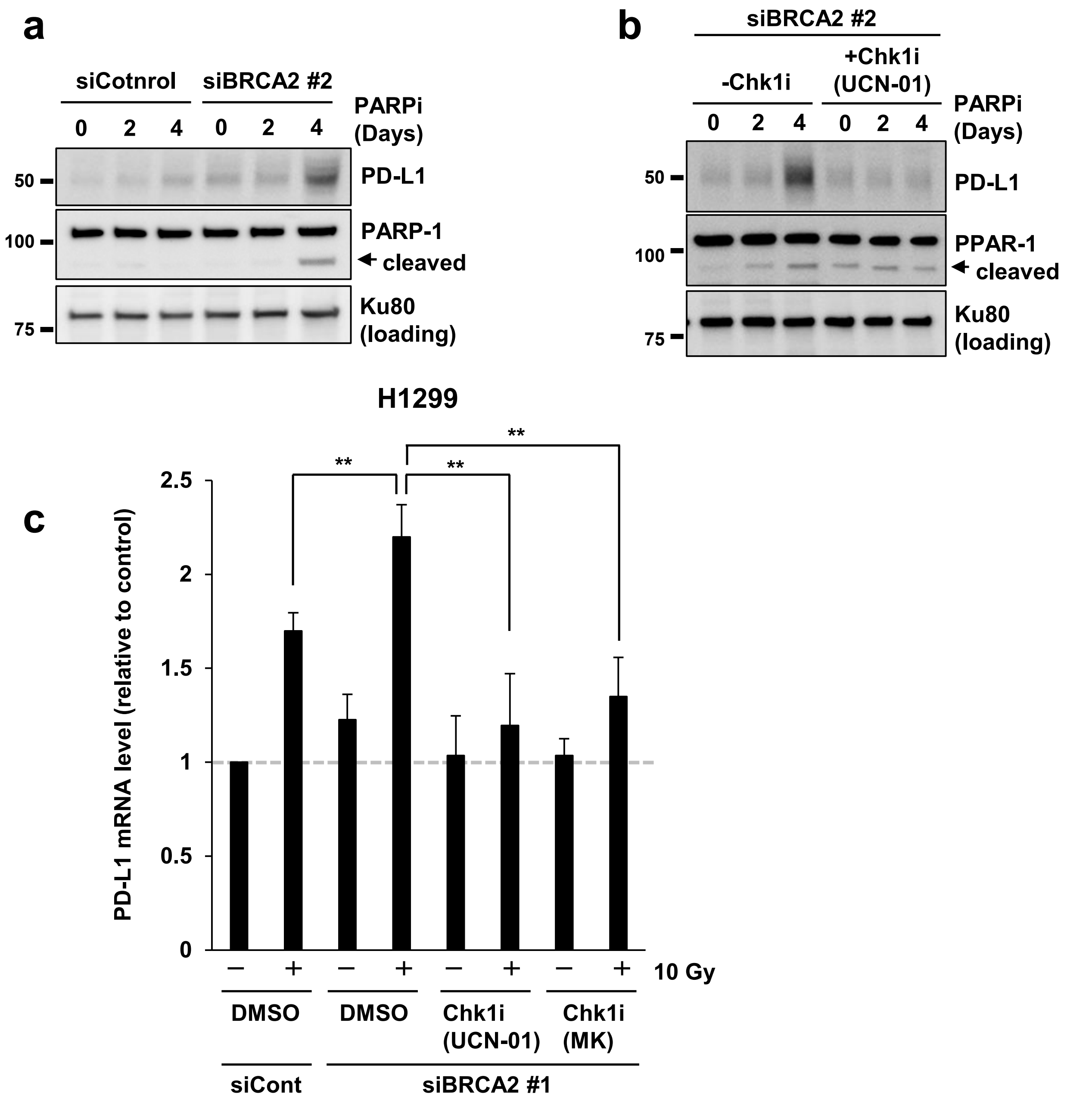

Supplementary Figure 6. Depletion of BRCA2 increases the upregulation of PD-L1 in response to IR or PARP inhibition

a) Similar to the data obtained by siBRCA2 #1 (Fig. 4b), depletion of BRCA2 by siBRCA2 #2 upregulates PD-L1 expression following the treatment with PARP inhibitor (PARPi). The knockdown efficiency of siBRCA2 #2 is shown in Fig. 4a.

b) The upregulation of PD-L1 in cells treated with siBRCA2 #2 + PARPi was suppressed by Chk1 inhibition.

c) Depletion of BRCA2 by siBRCA2 upregulates PD-L1 mRNA expression in H1299 cells after IR. The enhancement of PD-L1 mRNA was supressed by Chk1 inhibition. Cells were harvested 48 h after IR, and PD-L1 mRNA was measured by pPCR.

Error bars represent the s.d. of three independent experiments (c).

Supplementary Figure 7

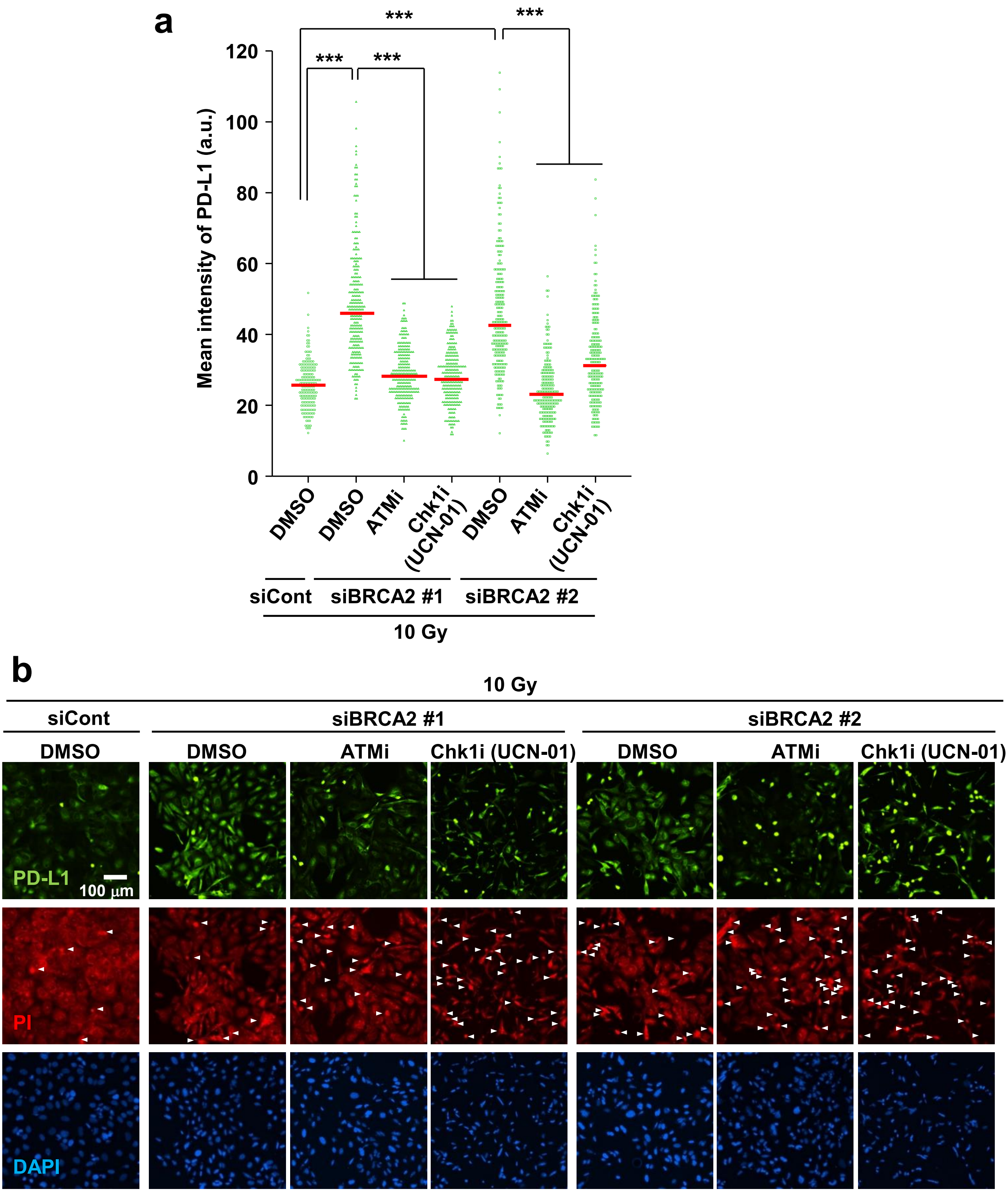

Supplementary Figure 7. Upregulation of cell-surface PD-L1 in BRCA2-depleted cells requires ATM/Chk1 activity

a) Cell-surface PD-L1 in BRCA2-depleted U2OS cells was examined by immunofluorescence without permeabilisation. Dead cells identified by PI were excluded from the analysis.

b) A representative image of panel a is shown.

Supplementary Figure 8

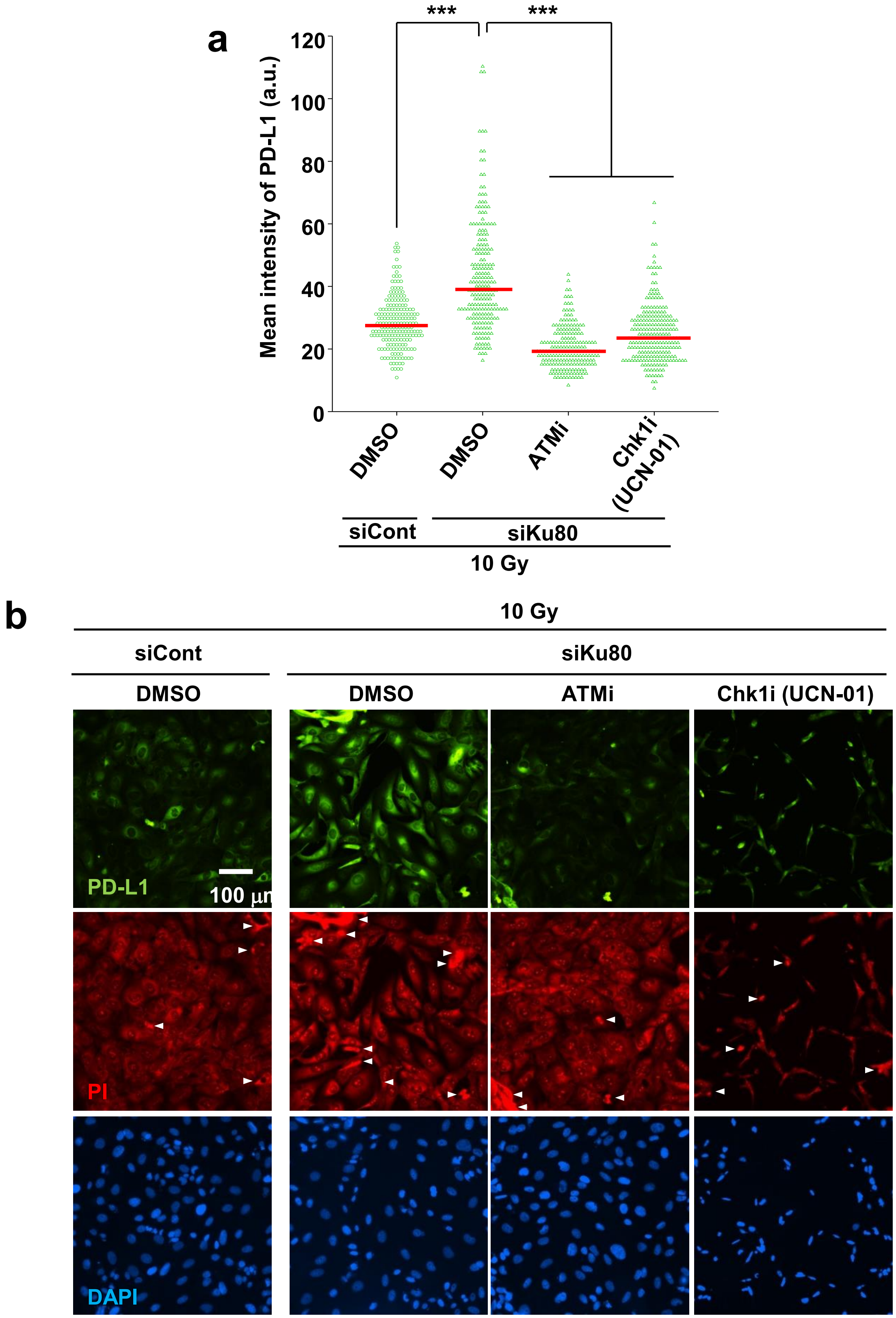

Supplementary Figure 8. Upregulation of cell-surface PD-L1 in Ku80-depleted cells requires ATM/Chk1 activity

a) Cell-surface PD-L1 in Ku80-depleted U2OS cells was examined by immunofluorescence without permeabilisation. Dead cells identified by PI were excluded from the analysis.

b) A representative image of panel a is shown.

Supplementary Figure 9

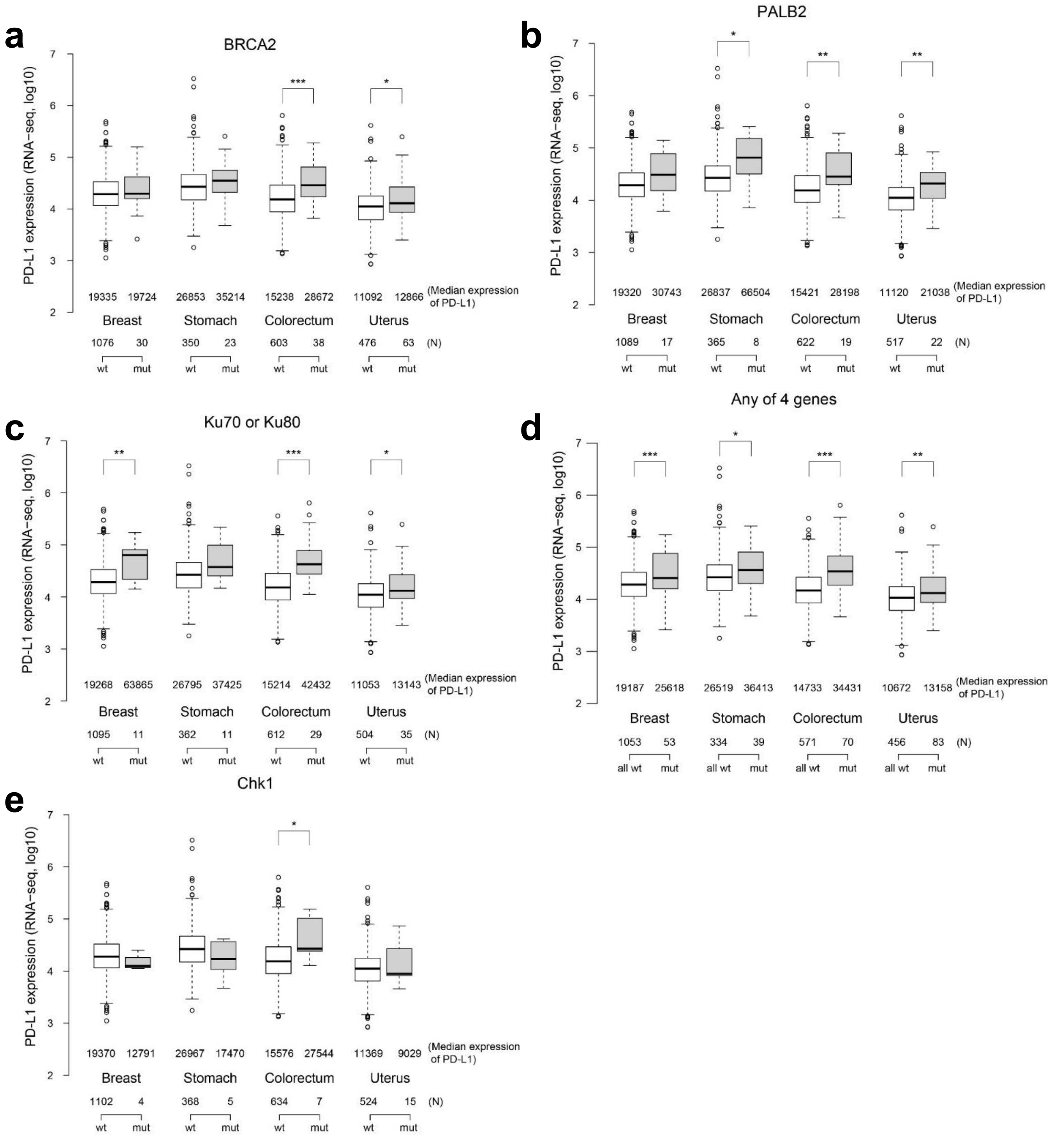

Supplementary Figure 9. Higher PD-L1 expression in tumours is associated with BRCA2, PALB2 and Ku70/80 mutations

a–c) PD-L1 expression levels in neoplastic breast, stomach, colorectal and uterine samples are shown regarding the mutation status in BRCA2 (a), PALB2 (b) and Ku70/80 (c). Analyses of other tumours are summarised in Extended Data Table 3.

d) Significant increases in PD-L1 expression in neoplastic breast, stomach, colorectal and uterine samples are observed in association with mutations in any of the four genes.

e) PD-L1 expression levels in neoplastic breast, stomach, colorectal and uterine samples are shown regarding the mutation status in Chk1.

Supplementary Figure 10

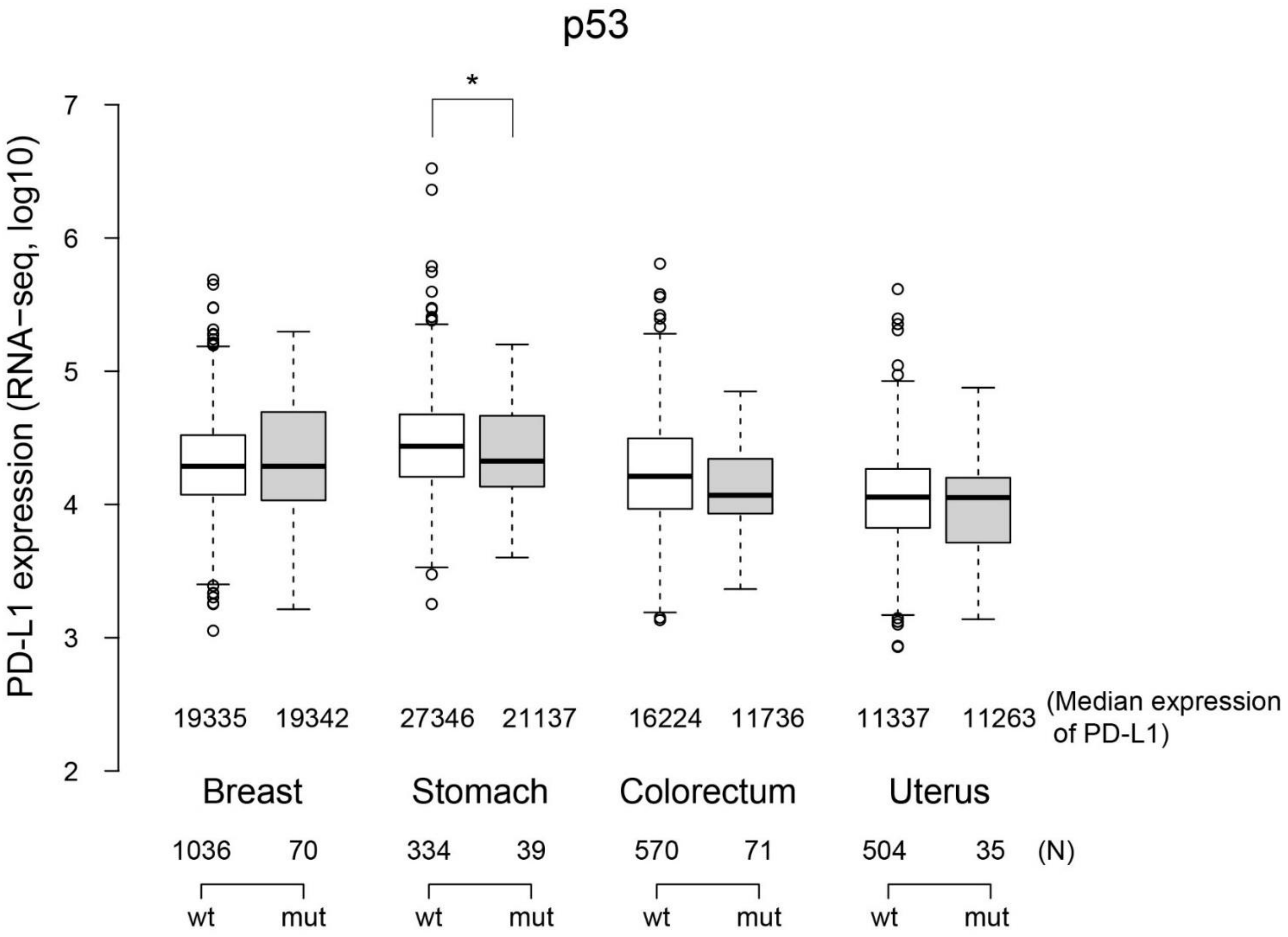

Supplementary Figure 10. PD-L1 expression in tumours is not associated with p53 mutation
There is not an obvious correlation between PD-L1 expression and p53 mutation. In stomach samples, p53 mutant shows a minor reduction of PD-L1 expression ($P < 0.05$).

Supplementary Figure 11

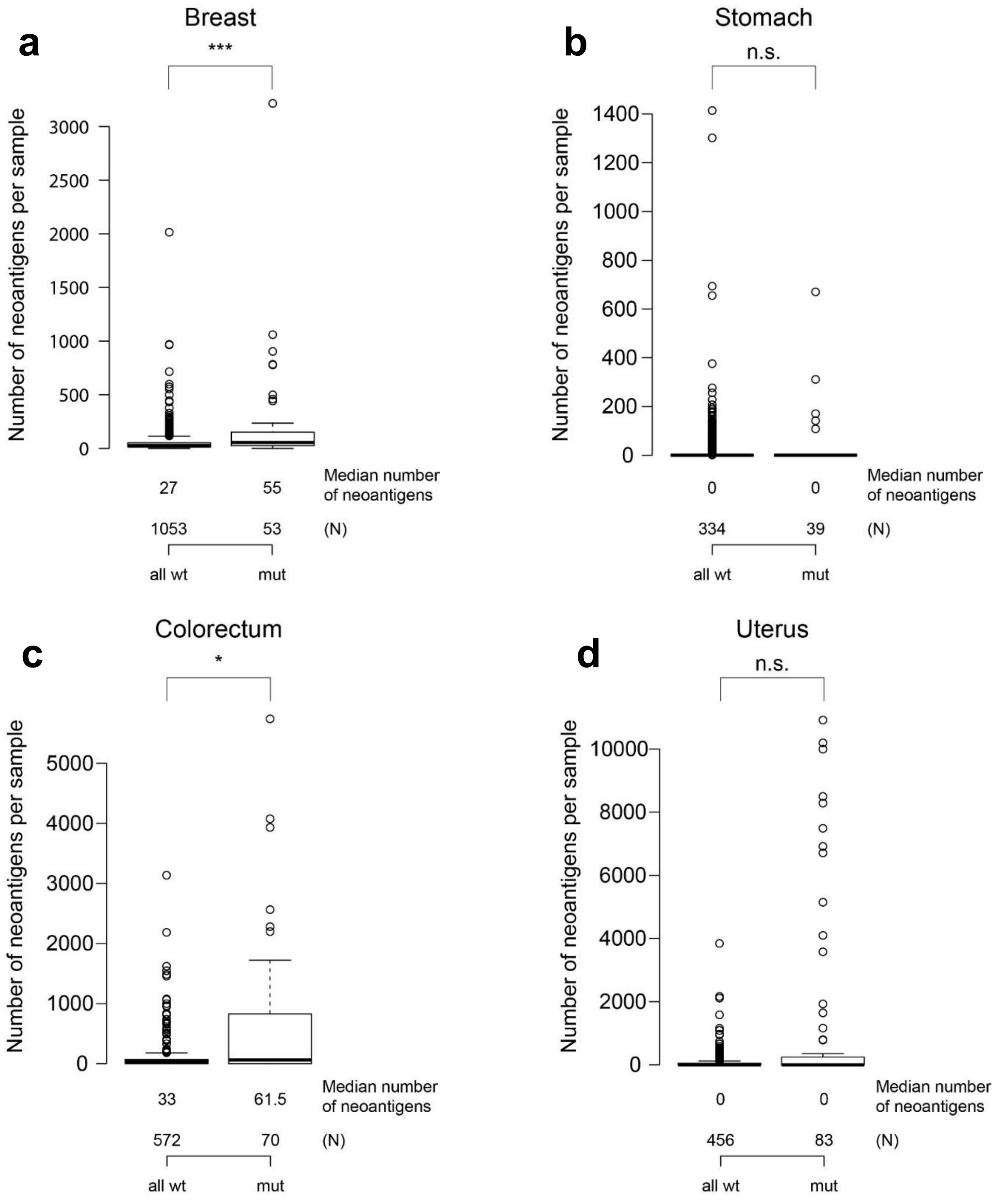

Supplementary Figure 11. Analysis of neoantigen levels in tumours harbouring mutation in DNA repair genes

a–d) The levels of neoantigens in neoplastic breast (a), stomach (b), colorectum (c) and uterus (d) samples are shown regarding the mutation status in any of the four genes, BRCA2, PALB2 and Ku70/80. While neoplastic breast and colorectal samples harbouring mutations in any of the four genes exhibit an increase in neoantigen levels, statistical significant difference was not observed in stomach and uterine samples.

Supplementary Figure 12

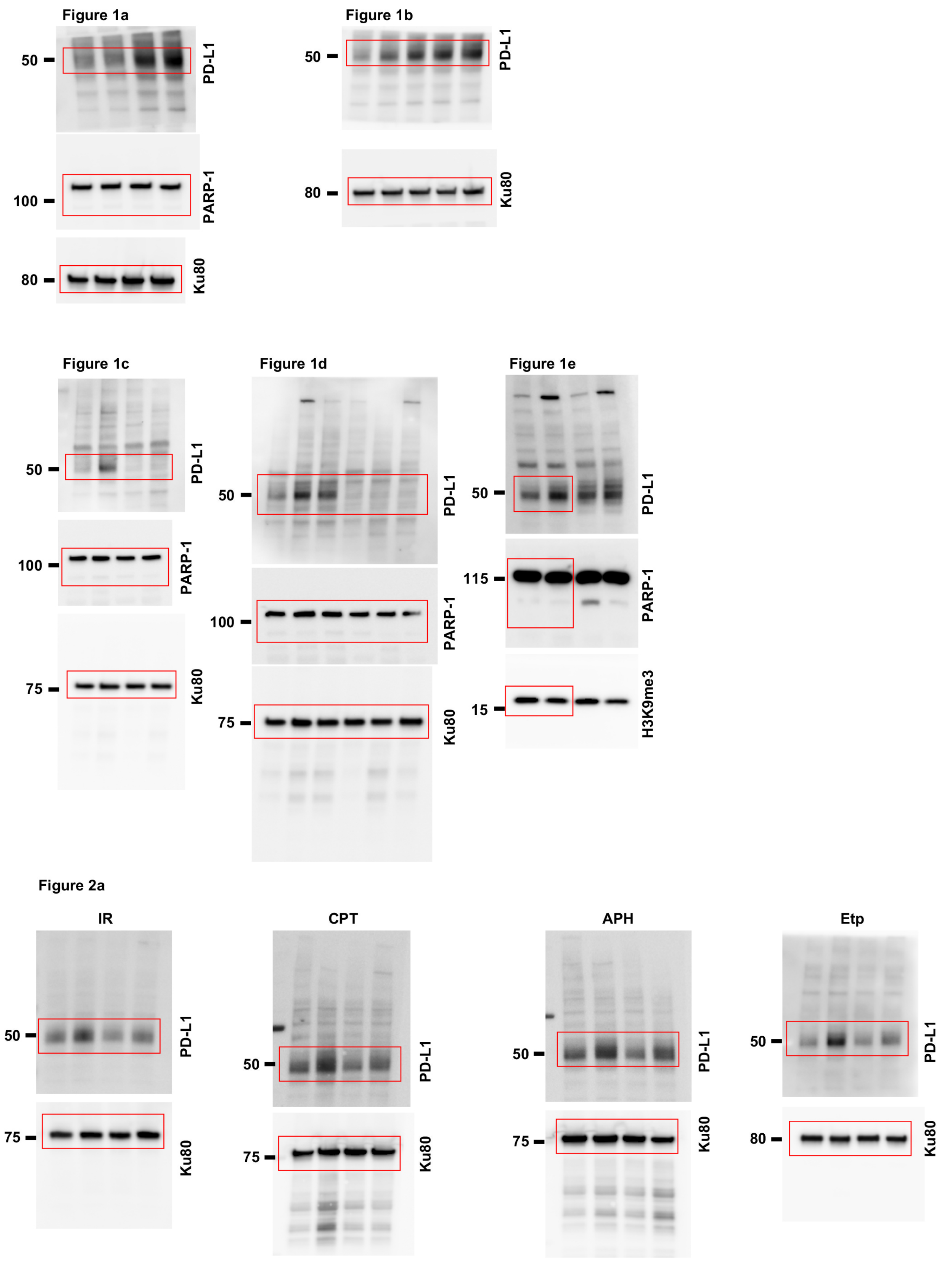

Figure 2b

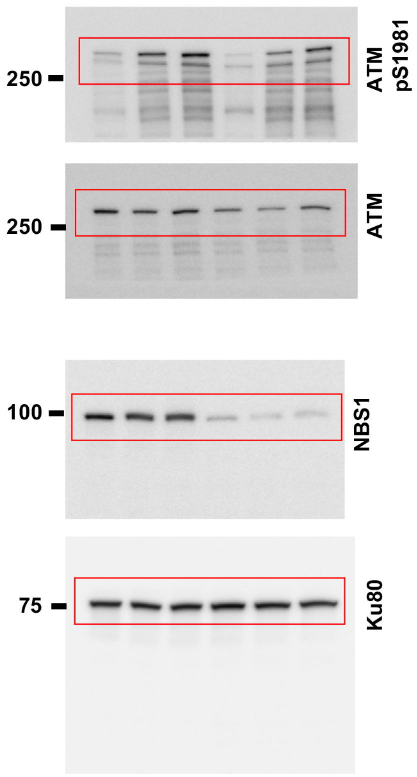

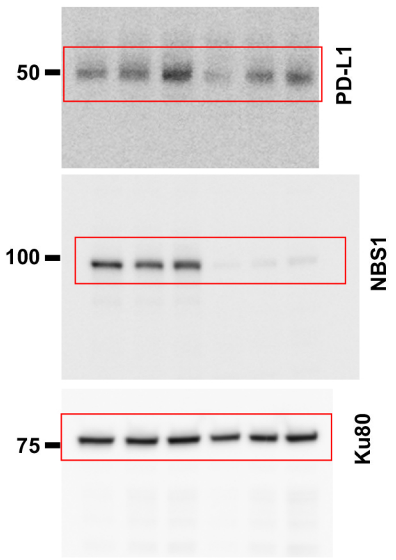

Figure 2d

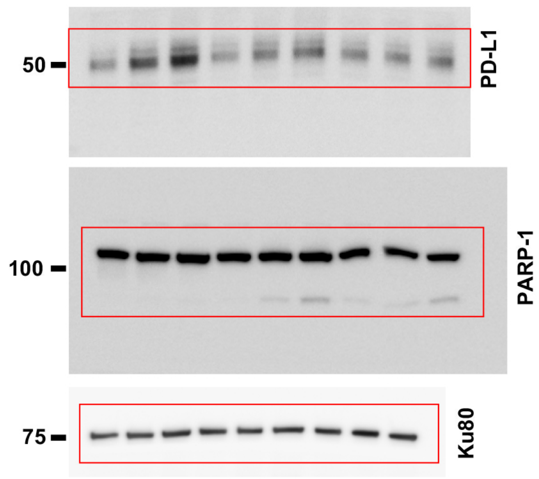

Figure 2e

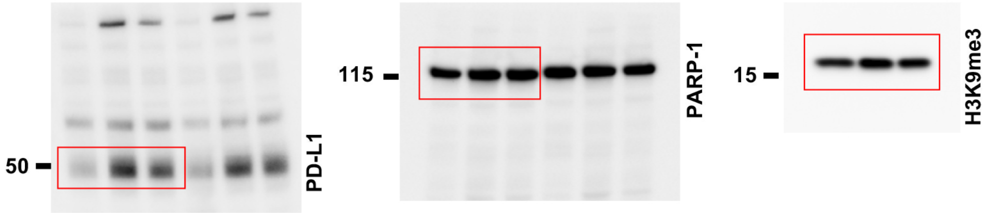

Figure 4a

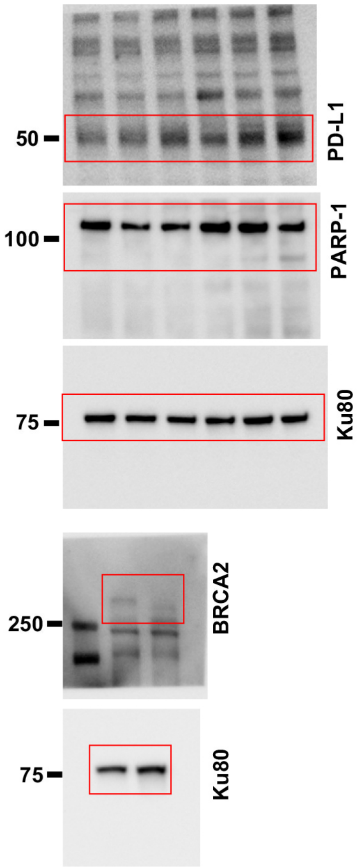

Figure 4b

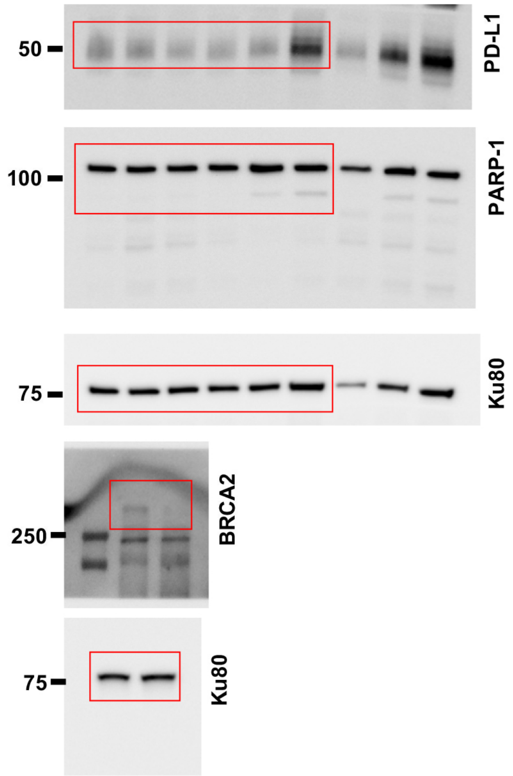

Figure 4c

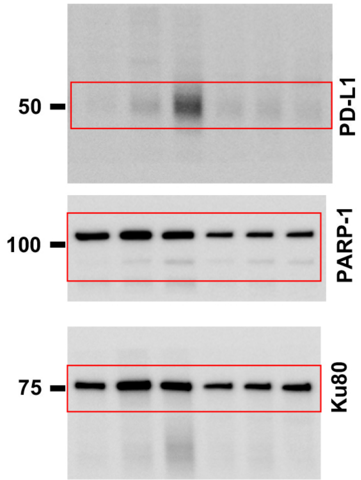

Figure 5a

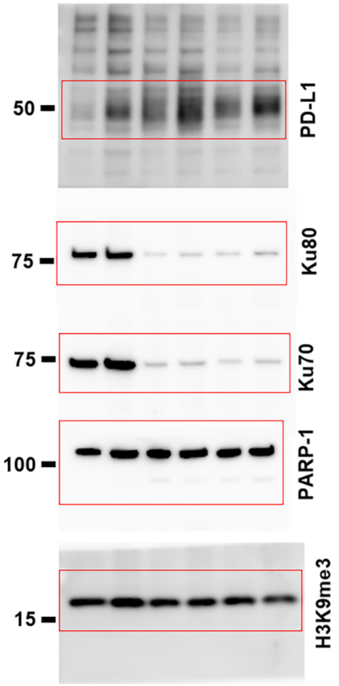

Figure 5b

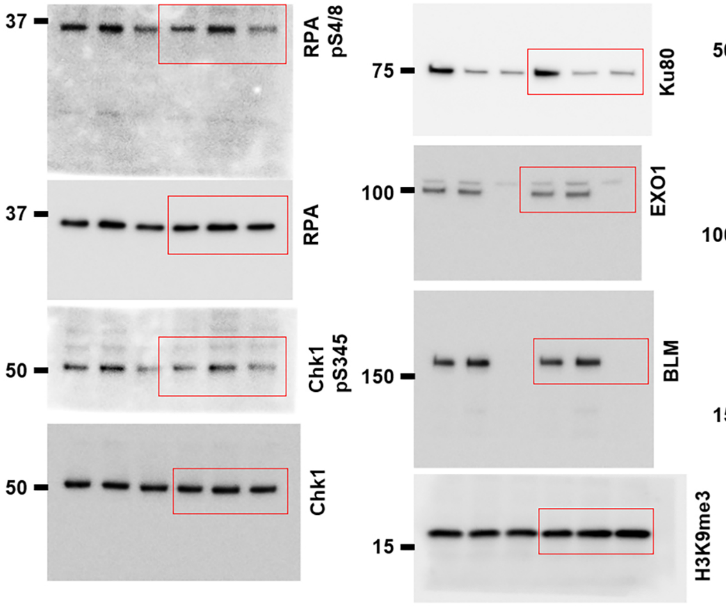

Figure 5c

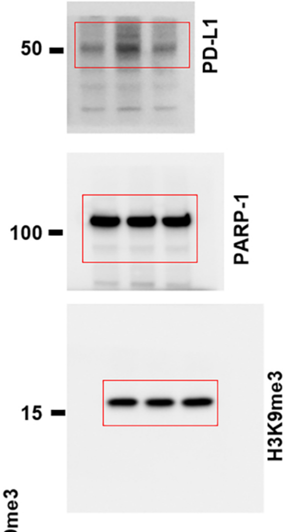

Figure 5d

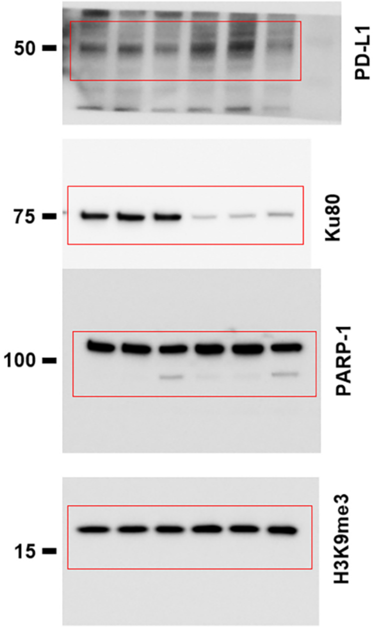

Figure 5e

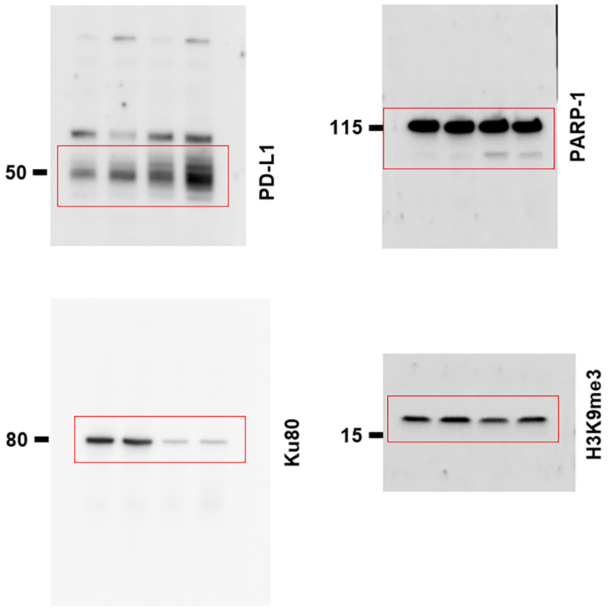

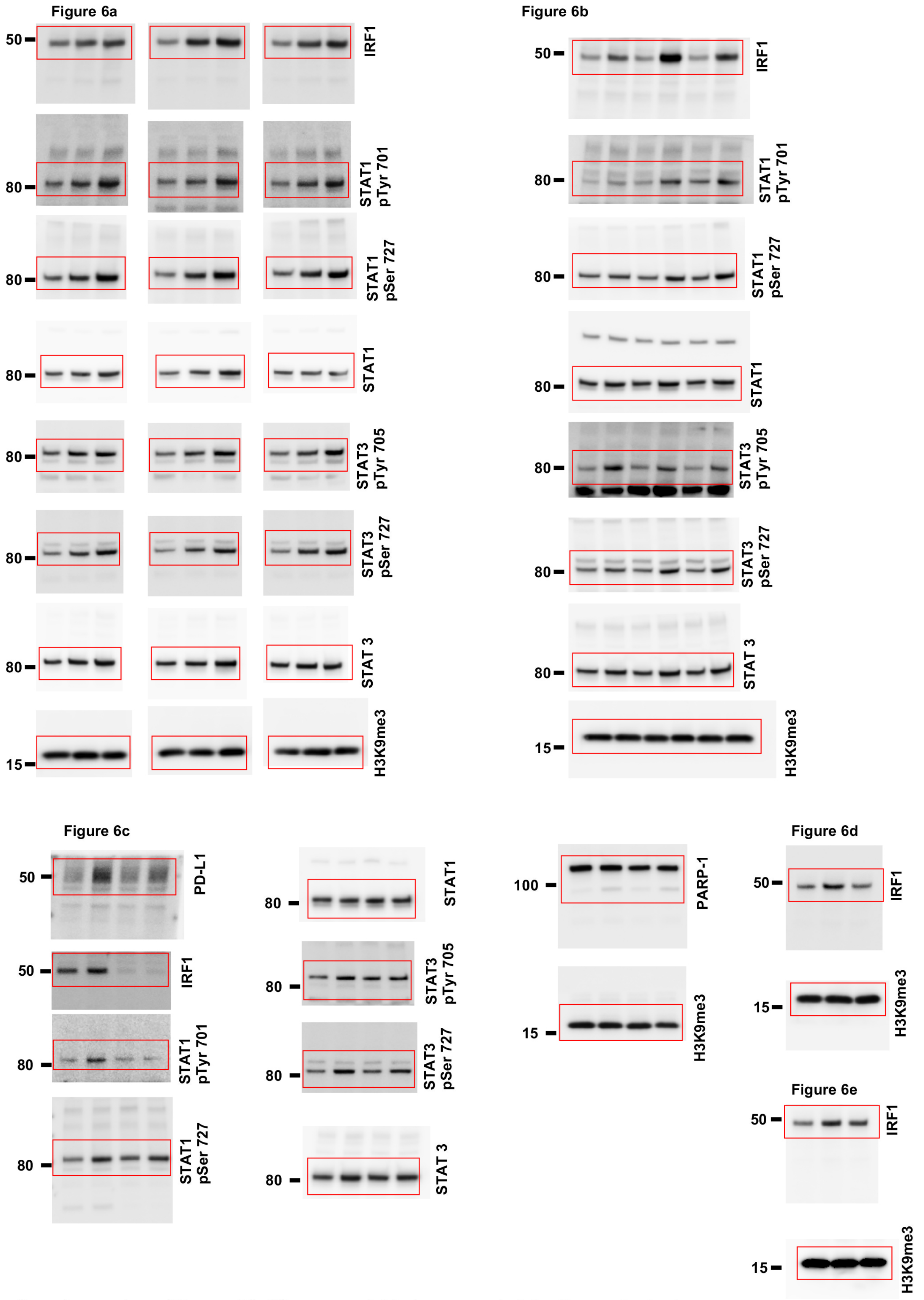

Supplementary Figure 12. Uncropped blots presented in the main text.

Supplementary Table 1. Summary of TCGA data set analysis

TCGA study	PD-L1 wt case N	Gene	Status	N	Median expression of PD-L1	PD-L1 expression ratio (mut/wt)	Mann-Whitney U test p-value	Significance
**BRCA**	1106	BRCA2	wt	1076	19335	1.02	0.2618	
			mut	30	19724			
		PALB2	wt	1089	19320	1.59	0.09590	
			mut	17	30743			
		Ku70/80	wt	1095	19268	3.31	0.002563	**
			mut	11	63865			
		Any	wt	1053	19187	1.34	0.0007147	***
			mut	53	25618			
**CESC**	304	BRCA2	wt	296	55351	0.46	0.1384	
			mut	8	25231			
		PALB2	wt	296	52442	0.98	0.9205	
			mut	8	51256			
		Ku70/80	wt	298	52442	1.80	0.3919	
			mut	6	94452			
		Any	wt	284	54043	0.59	0.9612	
			mut	20	32108			
**COAD+READ**	642	BRCA2	wt	604	15238	1.88	1.869E-05	***
			mut	38	28672			
		PALB2	wt	623	15421	1.83	0.001342	**
			mut	19	28198			
		Ku70/80	wt	613	15214	2.79	3.603E-08	***
			mut	29	42432			
		Any	wt	572	14733	2.33	1.454E-11	***
			mut	70	34431			
**HNSC**	501	BRCA2	wt	482	59770	0.57	0.1887	
			mut	19	34242			
		PALB2	wt	494	59412	0.57	0.7734	
			mut	7	33595			
		Ku70/80	wt	492	59294	1.09	0.9343	
			mut	9	64814			
		Any	wt	466	59769	0.62	0.2454	
			mut	35	37270			
**LUAD**	533	BRCA2	wt	506	52891	0.80	0.5479	
			mut	27	42378			
		PALB2	wt	516	51872	1.52	0.09425	
			mut	17	78943			
		Ku70/80	wt	513	52803	0.61	0.1238	
			mut	20	32174			
		Any	wt	471	52817	0.86	0.5363	
			mut	62	45194			
**LUSC**	500	BRCA2	wt	470	62157	0.80	0.7539	
			mut	30	49652			
		PALB2	wt	487	62592	0.61	0.1551	
			mut	13	38180			
		Ku70/80	wt	488	60710	2.33	0.05611	
			mut	12	141261			
		Any	wt	449	61849	0.95	0.5207	
			mut	51	58494			

Supplementary Table 1 (continued)

TCGA study	PD-L1 wt case N	Gene	Status	N	Median expression of PD-L1	PD-L1 expression ratio (mut/wt)	Mann-Whitney U test p-value	Significance
**SKCM**	458	BRCA2	wt	422	24744	0.83	0.4308	
			mut	36	20642			
		PALB2	wt	443	23497	1.56	0.1561	
			mut	15	36669			
		Ku70/80	wt	437	24725	0.76	0.5457	
			mut	21	18852			
		Any	wt	394	24706	0.92	0.8975	
			mut	64	22695			
**STAD**	373	BRCA2	wt	350	26853	1.31	0.3344	
			mut	23	35214			
		PALB2	wt	365	26837	2.48	0.03211	*
			mut	8	66504			
		Ku70/80	wt	362	26795	1.40	0.09538	
			mut	11	37425			
		Any	wt	334	26519	1.37	0.04789	*
			mut	39	36413			
**UCEC**	539	BRCA2	wt	476	11092	1.16	0.04036	*
			mut	63	12866			
		PALB2	wt	517	11120	1.89	0.005539	**
			mut	22	21038			
		Ku70/80	wt	504	11053	1.19	0.02707	*
			mut	35	13143			
		Any	wt	456	10672	1.23	0.002054	**
			mut	83	13158			

BRCA: Breast invasive carcinoma
CESE: Cervical squamous cell carcinoma and endocervical adenocarcinoma
COAD+READ: Colon adenocarcinoma + Rectum adenocarcinoma
HNSC: Head and Neck squamous cell carcinoma
LUAD: Lung adenocarcinoma
LUSC: Lung squamous cell carcinoma
SKCM: Skin Cutaneous Melanoma
STAD: Stomach adenocarcinoma
UCEC: Uterine Corpus Endometrial Carcinoma

Supplementary Table 2. List of siRNA used in this study

Name	Supplier	Sequence (sense 5'-3') or name of supplier
BLM	Dharmacon	ON-TARGETplus siRNA
BRCA2 #1	Dharmacon	ON-TARGETplus siRNA
BRCA2 #2	SIGMA (custom)	GAAGAAUGCAGGUUUAAUAdTdT
Control	SIGMA	GGGAUACCUAGACGUUCUAdTdT
EXO1	Dharmacon	ON-TARGETplus siRNA
IRF1	Dharmacon	ON-TARGETplus siRNA
Ku70	Dharmacon	ON-TARGETplus siRNA
Ku80	Dharmacon	ON-TARGETplus siRNA
NBS1	Dharmacon	ON-TARGETplus siRNA
PD-L1	Dharmacon	ON-TARGETplus siRNA
siRNA library	Dharmacon	ON-TARGETplus siRNA

Supplementary Table 3. List of antibodies and reagents used in this study

Target	Mono/polyclonal	Clone/reference	Antibody raised in	Source	Dilution for I.B.	Dilution for I.F.
ATM	Mono	D2E2	Rabbit	Cell Signaling Technology	1:1000	-
ATM pS1981	Mono	EP1890Y	Rabbit	Abcam	1:1000	-
BLM	Poly	Ab2179	Rabbit	Abcam	1:1000	-
BRCA2	Mono	2B	Mouse	Calbiochem	1:100	-
Chk1	Mono	2G1D5	Mouse	Cell Signaling Technology	1:500	-
Chk1 pS345	Mono	133D3	Rabbit	Cell Signaling Technology	1:500	-
EXO1	Poly	A302-640A	Rabbit	Bethyl Laboratories, Inc.	1:1000	-
H3K9me3	Poly	Ab8898	Rabbit	Abcam	1:4000	-
HLA	Mono	EMR8-5	Mouse	Hokudo Co., Ltd	1:20000	-
IRF1	Mono	D5E4	Rabbit	Cell Signaling Technology	1:1000	-
Ku70	Mono	N3H10	Mouse	Abcam	1:500	-
Ku80	Mono	C48E7	Rabbit	Cell Signaling Technology	1:4000	-
NBS1	Poly	PC269	Rabbit	Oncogene Research Products	1:1000	-
PARP-1	Poly	9542	Rabbit	Cell Signaling Technology	1:1000	-
PD-L1	Mono	E1L3N	Rabbit	Cell Signaling Technology	1:1000	1:200
RPA32	Mono	LS-C38952	Rat	LifeSpan BioSciences, Inc.	1:1000	-
RPA32 pS4/S8	Poly	A300-245A	Rabbit	Bethyl Laboratories, Inc.	1:1000	-
STAT1	Mono	D1K9Y	Rabbit	Cell Signaling Technology	1:1000	-
STAT1 pTyr701	Mono	58D6	Rabbit	Cell Signaling Technology	1:200	-
STAT1 pSer727	Mono	D3B7	Rabbit	Cell Signaling Technology	1:1000	-
STAT3	Mono	79D7	Rabbit	Cell Signaling Technology	1:1000	-
STAT3 pTyr705	Mono	D3A7	Rabbit	Cell Signaling Technology	1:1000	-
STAT3 pSer727	Mono	9134	Rabbit	Cell Signaling Technology	1:1000	-
IFN gamma	-	093-06111	-	Wako	-	-
Propidium iodide	-	PK-CA707-40017	-	PromoKine	-	1:2000

Minus signs indicate that the antibody was not used for this application in this study. I.B., immunoblotting; I.F., Immunofluorescence.

Antibody information for FACS	Source
APC Mouse IgG2b, κ Isotype Ctrl	Biolegend
APC anti-human CD274 (B7-H1, PD-L1)	Biolegend
